# A New Tool for Distinguishing Muscle Invasive and Non-Muscle Invasive Bladder Cancer: The Initial Application of Flexible Ultrasound Bronchoscope in Bladder Tumor Staging

**DOI:** 10.1371/journal.pone.0092385

**Published:** 2014-04-04

**Authors:** Chuanliang Xu, Zhensheng Zhang, Haifeng Wang, Qixiang Song, Rongchao Wei, Yongwei Yu, Jian Li, Yinghao Sun

**Affiliations:** 1 Department of Urology, Changhai Hospital, Second Military University, Shanghai, China; 2 Department of Biomedical Engineering, the Cleveland Clinic, Cleveland, Ohio, United States of America; 3 Department of Pathology, Changhai Hospital, Second Military University, Shanghai, China; 4 Department of Ultrasound, Changhai Hospital, Second Military University, Shanghai, China; Johns Hopkins University, United States of America

## Abstract

**Objectives:**

To validate the flexible ultrasound bronchoscope (FUB) as a tool in distinguishing muscle invasive and non-muscle invasive bladder tumors.

**Materials and Methods:**

From June 2010 to April 2012, 62 patients (11 female and 51 male) with 92 bladder urothelial carcinoma were treated in our study. The mean (±SD) patient age was 64.0±12.5 years old (ranged from 22 to 87). Clinical T stage was assessed by FUB at first in operating room, then immediately initial diagnostic transurethral resection (TUR) was performed. A second TUR would be done 2–4 weeks after initial TUR when the latter was incomplete (in large and multiple tumours, no muscle in the specimen) or when an exophytic high-grade and/or T1 tumour was detected. And radical cystectomy would be performed for the patients who were diagnosed with muscle-invasive tumors. FUB staging and initial TUR staging, final pathological results were compared.

**Results:**

In ultrasonic images, the normal muscle layer of bladder wall could be clearly distinguished into three layers, which were hyperechogenic mucosa, hypoechogenic muscle and hyperechogenic serosal. For non-muscle invasive tumors, the muscle layers were continuous. And distorted or discontinuous muscle layers could be seen in muscle-invasive case. The overall accuracy (95.7%) and the specificity of muscle invasion detection of FUB (98.8%) were comparable to TUR (overall accuracy 90.2% and specificity 100%), but sensitivity of muscle invasion detection of FUB was significantly higher than initial TUR (72.7%VS18.2%). Moreover, the tumor's diameter could not affect the FUB's accuracy of muscle invasion detection. For tumors near the bladder neck, FUB also showed the similar validity as those far from bladder neck.

**Conclusions:**

To conclude, the flexible ultrasound bronchoscope is an effective tool for muscle invasion detection of bladder tumor with ideal ultrasonic images. It is an alternative option for bladder tumor staging besides TUR. It might have the potentiality to change the bladder diagnostic strategy.

## Introduction

The clinical behavior, management strategy and prognosis for nonmuscle-invasive and muscle-invasive bladder cancer differ starkly, so accurate staging of bladder cancer is of great importance, especially distinguishing a nonmuscle-invasive tumor from a muscle-invasive tumor. However, computed tomography (CT) and magnetic resonance imaging (MRI) have limitations in distinguishing muscle-invasive and nonmuscle-invasive bladder tumors located in the bladder. They were more inclined to be used as tools to detect extravesical tumor involvement and lymph node metastasis [1], [2]. Although transurethral ultrasound and endoluminal ultrasound (ELUS) are good tools for the staging of bladder tumors that are confined to the bladder wall [3], they are limited by low resolution. To get better images with higher resolution, Horiuchi K used high-frequency ELUS for the staging of bladder tumors [4]. Unfortunately, high-frequency ELUS has a limited depth of penetration, which makes it difficult to evaluate the depth of invasion of tumors larger than 2 cm in size with a broad base [4]. In addition, ELUS is a rigid instrumentation and it can not provide good images for bladder tumors based in the bladder neck and intradiverticulum [5]. Thus, ELUS can not replace TUR, even though the muscle layer of the bladder is often absent in the specimen after transurethral resection of bladder tumor (TURBt), which is considered to be a significant clinical risk factor for understaging the tumor [6], [7]. Further more, the urethral ingury caused by ELUS examinations, especially for those old men with BPH, is another disadvantage.

To avoid the limitations and disadvantages of current tools for distinguishing between nonmuscle-invasive and muscle-invasive tumors (especially for larger tumors and tumors located near the bladder neck), we performed a pilot study that using a flexible ultrasound bronchoscope (FUB) to evaluate bladder tumors staging.

## Materials and Methods

### Patients and equipment

Men and women, aged >18 yr and with diagnosis of bladder cancer for the first time were eligible for inclusion. Patients with uretheral stricture, bladder stones, a previous history of baldder surgery, any psychiatric diseases, uncontrolled urinary tract infections, small bladder capacity (volume <100 mL), uncontrolled urinary tuberculosis, coagulopathy (such as hemophilia), and cardiovascular disease were excluded, and patients who could not tolerate the cystoscopy or CT scan or MRI were also excluded.

All the participants have provided their written informed consent in this study. This study was approved by the institutional review board of Changhai Hospital. Data were prospectively entered in a review board and reviewed retrospectively. June 2010 to April 2012, 62 patients were enrolled in our study.

**Figure 1:**
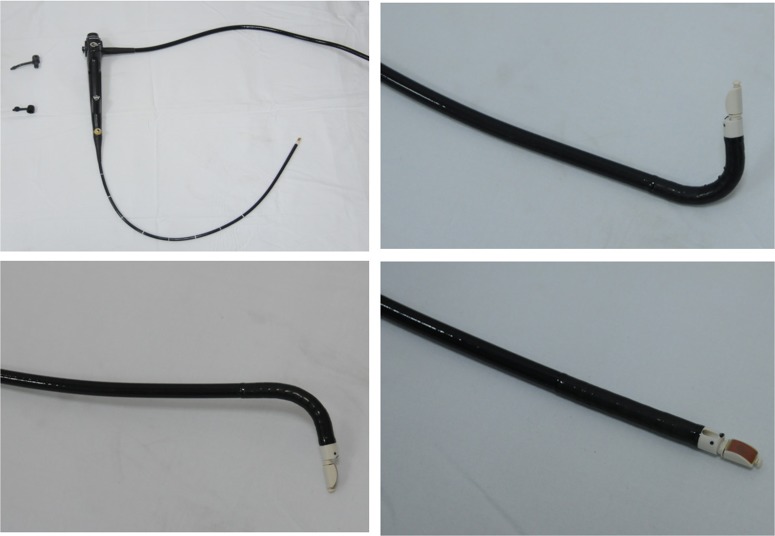
The flexible ultrasound bronchoscope.

All the patients were diagnosed as “bladder mass” by abdominal ultrasound examination. Before FUB examination, the patients had undergone intravenous pyelogram (to eliminate urothelial carcinoma of the ureter and renal pelvis), and a CT scan (to detect the extravesical tumor involvement and lymph node metastasis). The bladder examinations were performed with a FUB with a linear scanning transducer with quad frequencies (5, 7.5, 10, and 12 MHz) for depth of penetration of the ultrasound image on the tip (BF-uc260F-OL8; Olympus; Japan) (Figure 1). **Procedure**. The same operator who was skillful in performing flexible cystoscope carried out all the examinations. After general anesthesia, the FUB was inserted into the bladder under clear vision with the continual irrigation of saline into the bladder via a syringe. After examination of the entire bladder to confirm the location and number of the tumors, the ultrasound transducer was turned on. According to the direct endoscopic vision and simutaneous ultrasonic picture, the position of the probe could be adjusted in order to get a best ultrasound image at the proper angle and the closest position. For instance, if the tumor was larger than 2 cm, the probe could be put at the basement of the tumor; and if the tumor located at the anterior wall of the bladder, the probe would be flipped to touch the tumor. After obtaining the images which could clearly show us the continuous or distorted hypoechogenic bladder muscular layer, the conventional TUR was performed. A second TUR would be performed 2–4 weeks after initial TUR when the latter was incomplete (in large and multiple tumours, no muscle in the specimen), or when an exophytic high-grade and/or T1 tumour was detected according to EU guideline [8]. And radical cystectomy was performed for the patients who were diagnosed muscle-invasive tumors, or patients had extensive papillary disease that could not be controlled by TUR and intravesical therapy alone.

The FUB examination and data collection were done in accordance with the law of Chinese SFDA (state food and drug administration). Informed consent was also obtained from all patients.

### Index definition and statistical analysis

We compared the efficacy of FUB and other methods by several index as followed: “Overstage” meant making a diagnosis of “muscle invasive tumor” but the final pathological result indicated that it should be non-muscle invasive actually. On the contrary, “understage” meant making a diagnosis of “non-muscle invasive tumor” but the final pathological result indicated that it should be muscle invasive. “overall accuracy” meant the consistency of the prior diagnosis and the final pathological diagnosis. “Sensitivity of muscle invasion detection” measured the proportion of actual positive (muscle invasive) which were correctly identified as such. “Specificity of muscle invasion detection” measured the proportion of negatives(non-muscle invasive) which were correctly identified as such.

SPSS 11.5 for Windows was used for statistical analysis. Chi-Square test was used to determine differences of count data between groups,and P<0.05 was defined as the level of statistical significance.

## Results

In our study, 62 patients (11 women and 51 men, average age 64.0±12.5 years, range from 22 to 87) with 92 bladder urothelial carcinoma were enrolled. Of all 62 patients, solitary tumor was found in 38 patients and non-solitary tumor was found in other 24 patients. Second TUR was performed in 21 patients and radical cystectomy was performed in 13 paients. Of the 92 tumors, 11 were muscle invasive and 81 were non-muscle invasive according to the final pathological results. Dimension of the tumor was 2.3±1.2 cm, histologic results showed 44 tumors were G1, 35 were G2 and 13 were G3. CT scan were performed in 15 patients (with 20 tumors) and MRI were performed in the other 47 patients (with 72 tumors).

**Figure 2:**
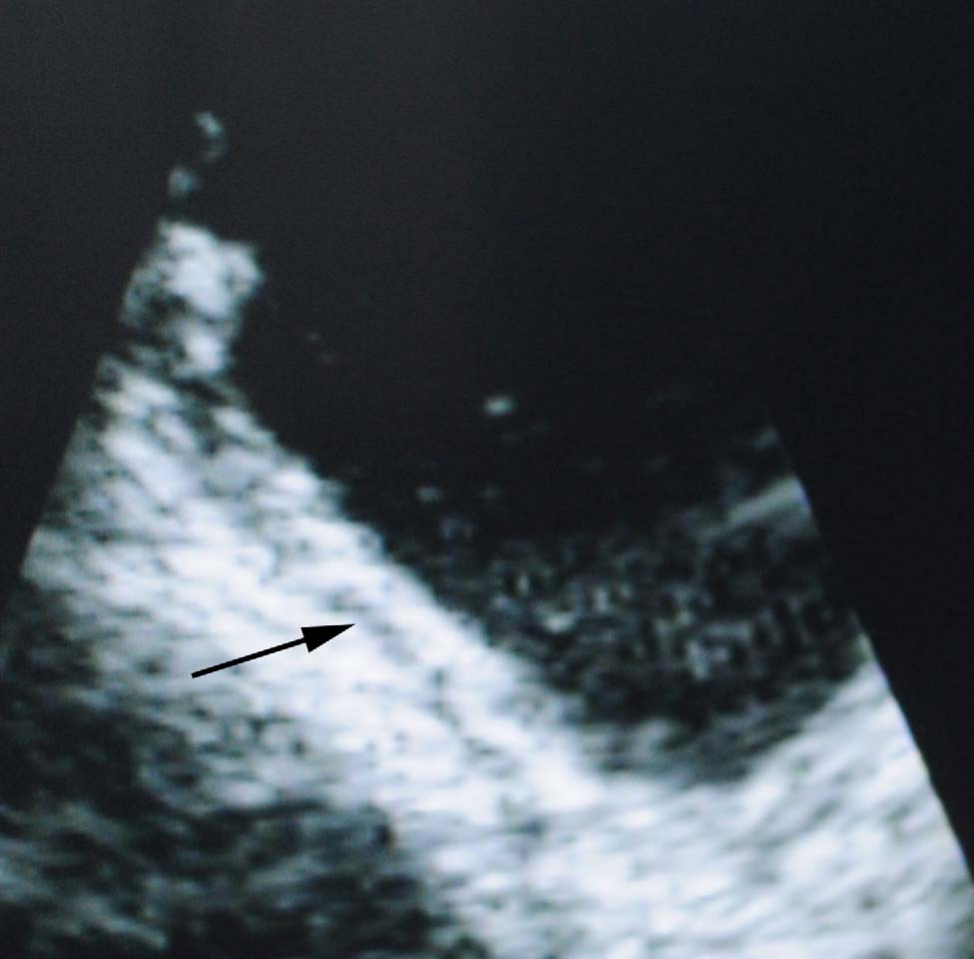
The normal muscle layer of bladder wall in the ultrasonic image.

**Figure 3:**
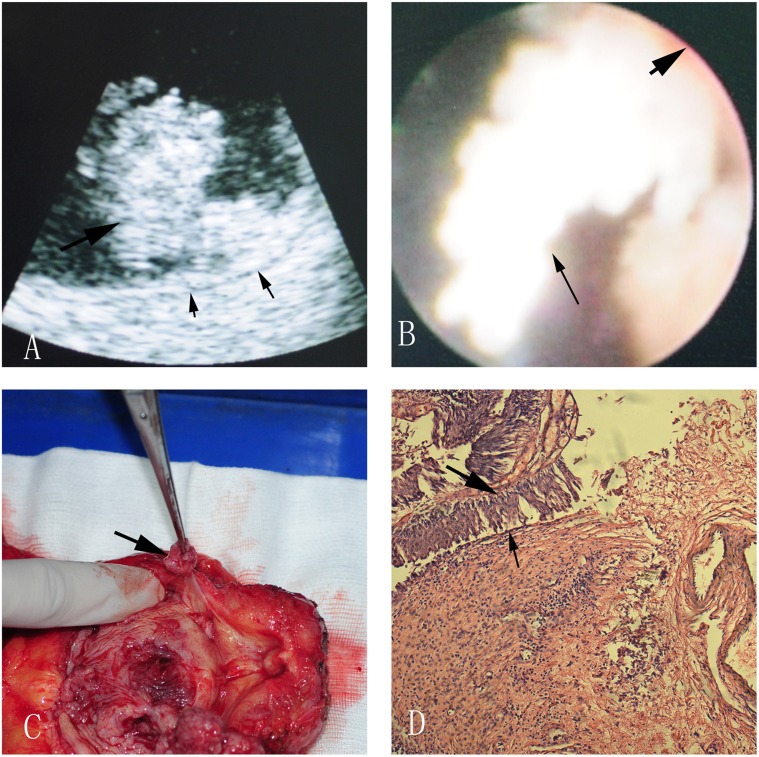
A nonmuscle-invasive bladder tumor near the bladder neck in ultrasonic image (A), direct version (B), radical cystectomy specimen (C) and pathological image (D). A: The big black arrow indicates bladder tumor, and the two small arrows indicate continuous muscle layer; B: The small black arrow points at the bladder tumor, and the big one points at the bladder wall; C: The arrow shows us a bladder tumor near the bladder neck; D: The big black arrow indicates bladder tumor, and the small arrow indicates normal bladder epithelium.

**Figure 4:**
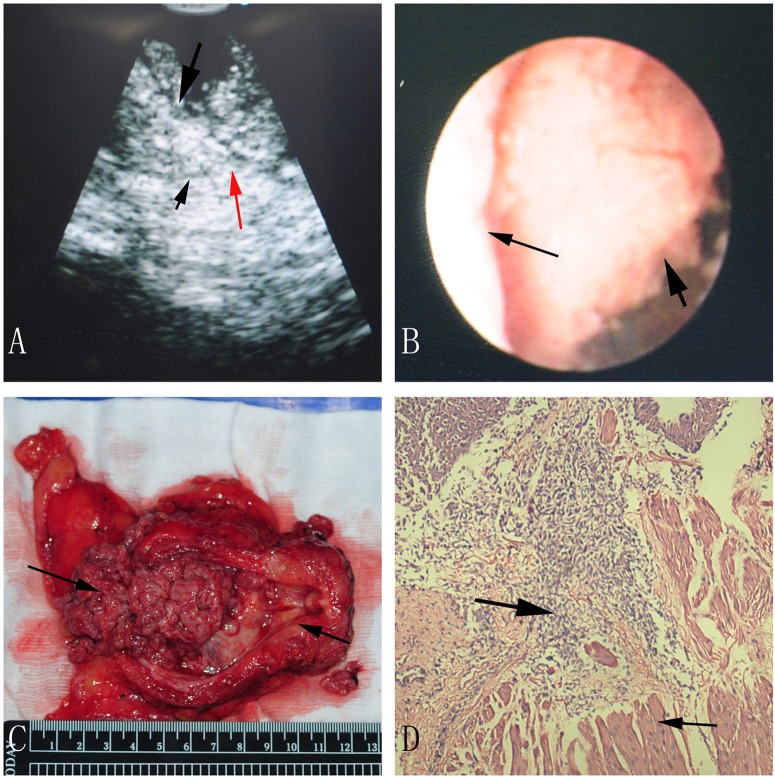
A muscle-invasive bladder tumor in ultrasonic image (A), direct version (B), radical cystectomy specimen (C) and pathological image (D). A: The big black arrow indicates bladder tumor, and the small black arrow indicates muscle layer, and the red arrow indicates uncontinuous muscle layer, and the blue arrow marked the bladder cavity; B: The big black arrow points at the bladder tumor, and the small one points at the bladder wall; C: The right arrow shows us a bladder tumor, and the left arrow shows us the bladder neck; D: The big black arrow indicates bladder tumor, and the small arrow indicates infiltrated muscle layer.

In ultrasonic images, the normal muscle layer of bladder wall could be clearly distinguished into three layers which were hyperechogenic mucosa, hypoechogenic muscle and hyperechogenic serosal (Figure 2). The muscle layers were continuous in nonmuscle-invasive case (Figure 3). Discontinuous muscle layers could be seen in muscle-invasive cases. In Figure 4, we could see a hypoechogenic layer marked by a small black arrow, and the hypoechogenic layer was disrupted by some hyperechogenic tissue which was marked by a red arrow. Figure 3 and Figure 4 showed a typical case with different subtypes of bladder tumor concurrently, one subtype was nonmuscle-invasive near the bladder neck (Figure 3) and the other one was muscle-invasive with the diameter larger than 4 cm (Figure 4).

When dividing the 92 tumors into two major categories: muscle invasive and non-muscle invasive, a good correlation was found between FUB and the final pathological results with a good overall accuracy. FUB correctly staged 95.7% (88/92) of tumors, overstaged 1.1% (1/92) and understaged 3.3% (3/92) of tumors (Table 1). Meanwhile, the initial TUR had a lower overall accuracy of 90.2% (83/92), and 9 tumors were understaged by initial TUR. The differrence of overall accuracy between FUB and TUR was not significant (p = 0.12). However, sensitivity of muscle invasive bladder cancer (MIBC) detection for FUB (72.7%(8/11)) was significantly higher than that of TUR (18.2%(2/11)), and specificity of MIBC detection for FUB (98.8%(80/81)) was comparable to TUR (100.0%(81/81)). (Table 1).

**Table 1:** Accuracy of tumor staging of FUB group and Initial TUR group.

		FUB (Total = 92)	Initial TUR (Total = 92)	Ultimate Pathologic stage (Total = 92)	*P* value
MIBC	Subtotal	9	2	11	-
	Overstage	1	0	-	-
	Consitency	8	2	-	
NMIBC	Subtotal	83	90	81	-
	Understage	3	9	-	-
	Consitency	80	81	-	-
Overall consitency		88(8+80)	83(2+81)	-	-
Overall accuracy		95.7%(88/92)	90.2%(83/92)	-	0.12
Sensitivity of MIBC Detection		72.7%(8/11)	18.2%(2/11)	-	0.02
Specificity of MIBC Detection		98.8%(80/81)	100%(81/81)	-	1.00

MIBC:Muscle invasive bladder cancer.

NMIBC:Non-muscle invasive bladder cancer.

There were 8 tumors with the diameter larger than 4 cm, and 84 tumors smaller than 4 cm. Though the sample size was small, it could be found that the tumor's diameter could not affect the overall accuracy of clinical staging (87.5% to 98.8%) (Table 2).

**Table 2:** Stage estimation for tumors of different size.

		Larger than 4 cm (Subtotal = 8)		Smaller than 4 cm (Subtotal = 84)		*P* value
		FUB	Ultimate Pathologic stage	FUB	Ultimate Pathologic stage	
MIBC	Subtotal	3	4	6	5	-
	Overstage	0	-	1	-	-
NMIBC	Subtotal	5	4	78	79	-
	Understage	1	-	0	-	-
Overall accuracy		87.5%(7/8)	-	98.8%(83/84)	-	0.82
Sensitivity of MIBC detection		75.00%(3/4)	-	100%(5/5)	-	0.91
Specificity of MIBC detection		100%(4/4)	-	98.7%(78/79)	-	0.82

MIBC:Muscle invasive bladder cancer.

NMIBC:Non-muscle invasive bladder cancer.

When dividing the tumors into 2 subgroups according to the tumors location in the bladder, we found that overall accuracy of FUB was comparable to TUR for tumors either near the bladder neck (90.0% VS 81.8%) or far from the bladder neck(96.3% VS 91.4%) (Table 3).

**Table 3:** Stage estimation for tumors at different locations.

		Near the bladder neck (Subtotal = 11)				Far from the bladder neck (Subtotal = 81)			
		FUB	Initial TUR	Ultimate Pathologic stage	*P* value	FUB	Initial TUR	Ultimate Pathologic stage	*P* value
MIBC	Subtotal	3	2	4	-	6	0	7-	-
	Overstage	0	0	-	-	1	-	-	-
	Consitency	3	2	-	-	5	-	-	
NMIBC	Subtotal	8	9	7	-	75	81	74	-
	Understage	1	2	-	-	2	7	-	-
	Consitency	7	7	-	-	73	74	-	
Overall consitency		10	9	-	-	78	74	-	
Overall accuracy		90.0% (10/11)	81.8% (9/11)	-	0.53	96.3% (78/81)	91.4% (74/81)	-	0.11

MIBC:Muscle invasive bladder cancer.

NMIBC:Non-muscle invasive bladder cancer.

CT scan were performed in 15 patients (with 20 tumors) and MRI were performed in the other 47 patients (with 72 tumors). The overall staging accuracy of CT, MRI and FUB was 55.0% (11/20), 61.1% (44/72) and 95.7% (88/92) respectively. When compared the MRI and CT, FUB could achieve more accurate muscle invasion detection (Table 4).

**Table 4:** Stage estimation by MRI and CT.

		MRI (Subtotal = 72)	CT (Subtotal = 20)	FUB (Total = 92)	P value	Ultimate Pathologic stage
MIBC	Subtotal	36	12	9	-	11
	Overstage	28	9	1	-	-
NMIBC	Subtotal	36	8	83	-	81
	Understage	0	0	3	-	-
Overall accuracy		61.1%(44/72)	55.0%(11/20)	95.7%(88/92)	both<0.001	-

MIBC:Muscle invasive bladder cancer.

NMIBC:Non-muscle invasive bladder cancer.

*P* value: Comparison of overall accuracy between MRI and FUB stage (61.1% vs 95.7%),and between CT and FUB stage(55.0% vs 95.7%).

## Discussion

Many types of examinations have been used in recent years to achieve accurate clinical staging for the optimum treatment of patients with urothelial carcinoma of the bladder. None of the available modalities, however, can provide accurate information on the depth of tumor invasion [4]. Although the dynamic MRI seemed to have the potential to be an effective tool in bladder cancer staging [9], a recent study about MRI and CT in staging of bladder cancer showed that MRI and CT only correctly staged 56% and 50% of tumors respectively [10]. Even in the optimistic article, the overall accuracy of MRI could only reach 76% [11]. Thus, for many years, TUR was considered to be the gold standard for the tumor staging, however, incidents of incomplete resection with clinical understaging of bladder tumors were widespread [12]. Acturally, the understaging rate was 3.4–20.6% according to Brausi's study [13]. Miladi et al also reported that the tumor stage was underestimated in 9–49% of TUR lesions [14].

The normal bladder wall can be divided into three layers under ultrasonography (i.e., hyperechogenic mucosa, hypoechogenic muscle and the hyperechogenic serosal) [5], and several ultrasonic techniques (i.e., transabdominal, transrectal and transurethral ultrasonography) have been used in the staging of bladder tumors [15], [16]. Because transabdominal and transrectal ultrasonography have similar advantages and disadvantages with CT and MRI in the bladder tumors staging, they can only be used as tools to detect extravesical involvement, and can not be used to detect the degree of bladder wall invasion [15]. With the development of endoscopy tools and techniques, transurethral ultrasonography can give useful information about bladder wall invasion. Transurethral ultrasonography, however, is not flexible for efficient adjustments of the probe position, which can cause complications like bladder and prostate injury. In addition, transurethral ultrasound is useless for the staging of tumors located near the bladder neck [16]. A study in 1980 utilized an ultrasonic endoscope to perform gastroscopy, and found that this new tool could allow both endoscopic visualization of the upper gastrointestinal tract and ultrasonic scanning of internal organs next to the gastrointestinal tract in one examination [17]. Since that time, the ultrasonic endoscope has been widely used in the staging of gastric cancer [18] and lung cancer [19]. And soon ELUS became the typical ultrasonic endoscope that was used in bladder tumor diagnosis. Alhough ELUS has the advantage of providing both endoscopic visualization and ultrasonic scanning, ELUS has not been widely used for several reasons. First, high frequency ELUS can give better resolution, but it can not evaluate the depth of invasion of tumors larger than 2 cm in size with a broad base [4]. Secondly, assessing the degree of the bladder wall invasion for tumors located in the bladder neck or the intradiverticulum is difficult with ELUS [5].

To overcome these shortcomings, we investigated the use of a FUB in the diagnosis of bladder tumors. After analyzing the data of this study, we found the FUB was an ideal tool for distinguishing muscle-invasive and nonmuscle-invasive tumors. Compared with rigid endoscopy, the FUB had a more facile probe, which made it possible to get clearer ultrasonic images because the tumor could be scanned at a more suitable angle and a more rational position. In addition, the flexibility of the bronchoscope and the simutaneous visualization when passing through the urethral could reduce the injury of urethral maximumly. Taken together, the results of the present study suggested that the use of a FUB for tumor staging could be an ideal tool for distinguishing between muscle-invasive and nonmuscle-invasive bladder tumors because it resulted in clearer ultrasonic images and less injuries.

According to our data, considering the final pathological results of the second TUR and radical cystectomy as the real tumor staging criteria, the accuracy of FUB stage was better than initial TUR stage (Table 1). The result might due to the following two reasons: (1) Limited by the experiences of surgeon and the TUR technique itself, some studies had showed that TUR specimens did not include the detrusor muscle which was invaded by the tumors [12], [13], [14]. While FUB could scan the basement of tumors all-round (due to the flexible probe, we could scan the tumors at any angle) and provide real-time clear ultrasound images. The statement of the tumor basement could be more clearly noticed than TUR. (2) Because TUR is a destructive surgery, the tissue resected from bladder might be destroyed, and the fact that muscle layer has been invaded by tumor might be omitted. Besides, pathologist's subjective errors also could cause false negative. Fortunately, FUB could clearly show us the structure of the bladder wall without any damage, and be affected by little subjective factors.

Interestingly, the staging accuracy of the FUB for tumors larger than 4 cm was the same as the accuracy for smaller tumors (Table 2). That was to say, FUB could overcome the disadvantage of ELUS in staging larger tumors [4], and it was an ideal staging tool for various size tumors. The improvement compared with the ELUS was due to the flexible probe, which could be moved to the base of the tumor or be adjusted to a suitable distance from the tumor at an appropriate angle. And due to the same reason, the FUB had similar accurate rate for tumors near the bladder neck and far from the bladder neck (Table 3). So FUB could overcome the limitations of ELUS in diagnosing tumors near the bladder neck.

Because some T1 tumors diagnosed by initial TUR were actually T2 [12], [13], [14], the FUB might be useful for urologists to make a decision on when to abandon the conservative therapy and remove the bladder without restaging,especially be helpful for the patients who might be diagnosed as “T1” tumors by initial TUR.

## Conclusions

In conclusion, the FUB is a potentially effective tool for staging bladder tumors and provides clear ultrasonic images while reducing urethral injuries. With the accumulation of clinical data, we might build a new diagnostic strategy for distinguishing muscle-invasive and nonmuscle-invasive bladder tumors. Cruder outside diameter than ordinary flexible cystoscope, limited application cases and less clear direct version are needed to be improved. The inability in distinguishing Ta and T1 tumors is its disadvantage. And the accuracy of FUB for staging tumors located in the intradiverticulum needs further evaluation.
